# Mediation Analysis in Discipline-Based Education Research Using Structural Equation Modeling: Beyond “What Works” to Understand How It Works, and for Whom

**DOI:** 10.1128/jmbe.00108-21

**Published:** 2021-06-30

**Authors:** Cissy J. Ballen, Shima Salehi

**Affiliations:** a Department of Biological Sciences, Auburn University, Auburn, Alabama, USA; b Graduate School of Education, Stanford University, Stanford, California, USA

**Keywords:** mediation analysis, discipline-based education research, SEM, biology education research, methods

## Abstract

Advancing the field of discipline-based education research (DBER) requires developing theories based on outcomes that integrate across multiple methodologies. Here, we describe mediation analysis with structural equation modeling as one statistical tool that allows us to further examine mechanisms underlying well-documented trends in higher education. The use of mediation analysis in educational settings is particularly powerful, as learning outcomes result from complex relationships among many variables. We illustrate how mediation analysis can enhance education research, addressing questions that cannot be easily reached otherwise. We walk through critical steps to guide decision-making in mediation analysis and apply them to questions using real data to examine performance gaps in large introductory courses in biology. Through the use of mediation analysis with structural equation modeling, we add to a growing body of research that shows diverse quantitative approaches support evidence-based teaching in higher education.

## PERSPECTIVE

Understanding the underlying mechanisms for learning is a goal for education researchers. Why do we observe learning gains in response to an activity? Why do different teaching approaches benefit one student population but fail to generalize across other populations? What are the mechanisms through which strategies help or hinder students? While a regression model shows the relationship between one or more independent variables and a single dependent (outcome) variable, it rarely describes how variables transmit their effects on the outcome of interest (1). This means that other statistical methods should be explored as better strategies to address complex analytical questions. One such strategy is mediation analysis.

Mediation analysis explores a chain of relationships between a number of variables. This analysis, while common in other social science fields, is less common in STEM fields, in which many individuals who conduct discipline-based education research (DBER) are skilled. DBER is a term used to describe research in the context of higher education that has firm grounding within a specific discipline (2). There are many disciplinary differences among scientists in the natural sciences (which focus on biology, chemistry, and physics) and in the social sciences (which focus on human dimensions of the world). Each broad discipline approaches research in different ways, each with their own methodology, terminology, and culture. Mediation analysis is an example of an analytical tool that is used less in the natural sciences than the social sciences. For those who lack a familiarity due to a natural science background, learning mediation analysis can seem daunting. However, it is highly relevant to DBER fields that use experimental and observational data to explore the relationship between many interconnected variables and answer questions about teaching and learning. While previous work walks through other common statistical methods in the context of DBER, such as method papers that describe factor analysis (3), generalized linear models (4), and the use of random effects in multilevel regression models (5), no such how-to guide exists for individuals applying mediation methods to DBER.

**FIG 1:**
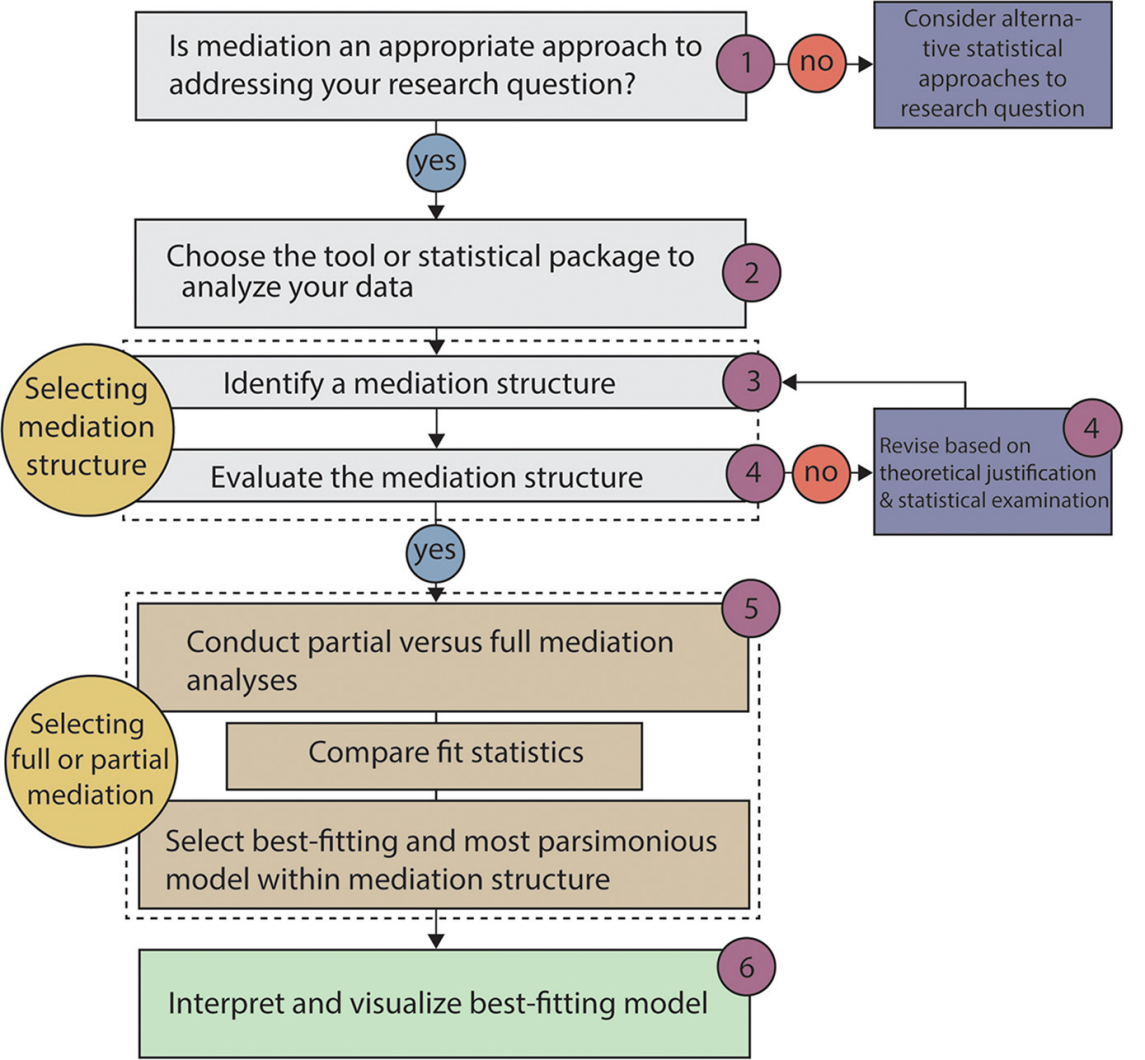
Flowchart of critical steps to guide decision-making in mediation analysis. Numbers correspond to sections in the manuscript where we elaborate with considerations and instructions for completing each step. We provide an extended example illustrating this process, along with R code, in the last section.

Our guide to decision-making in mediation analysis has the following main stages (Fig. 1). (i) We build a case for mediation analysis and describe common reasoning behind using mediation analysis in DBER. (ii) We explain why structural equation modeling is a useful tool with which one can conduct mediation analysis, with a focus on using the *lavaan* package in R for structural equation modeling. (iii) We discuss how to select a mediation structure, consider different statistical models, and assess the fit of statistical models to the data (Table 1). (iv) We discuss how to run a model and report the results. We then provide an example from the context of biology classrooms that illustrates the use of mediation analysis, which can be found in the supplemental material. Our hope is that readers gain new insights into analyzing data, even revisiting those that they already possess, by looking at them through a mediation lens.

**TABLE 1:** Summary of fit indices and the desired outputs in mediation analysis

Fit index	What is measured	Rule(s) of fit
χ^2^	Determines the magnitude of discrepancy between the covariance matrix estimated by the model and the observed covariance matrix of the data sets	Should be nonsignificant, meaning the estimated covariates are not significantly different from the actual data covariates
Comparative fit index (CFI)	Determines if the model fits the data by comparing the χ^2^ of the model with the χ^2^ of the null model; adjusts for sample size and no. of variables	>0.90, acceptable; >0.95, good
Root mean square error of approximation (RMSEA)	Determines how well the model fit the data and favor parsimony and a model with fewer parameters	<0.05 to 0.06, good; 0.06 to 0.08, acceptable; 0.08 to 0.10, mediocre; >0.10, unacceptable
Standardized root mean square residual (SRMR)	A standardized square-root of the difference between the observed correlation and the predicted correlation	< 0.05, good; 0.05 to 0.08, acceptable; 0.08 to 0.10, mediocre; >0.10, unacceptable
Akaike information criterion (AIC)	Determines if one model fits the data better than the other	The lower value is preferred when comparing two model estimations from the same data set
Baysian information criterion (BIC)	Determines if one model fits the data better than the other; while AIC has a penalty of 2 for every estimated variable, the BIC penalty increases with an increase in sample size	The lower value is preferred when comparing two model estimations from the same data set
Tucker Lewis index (TLI)	Determines to what extent the model of the interest improves the fit compared to the fit of the null model	TLI ≥ 0.95

## MEDIATION ANALYSIS

**FIG 2:**
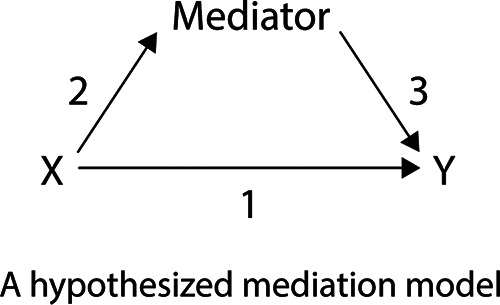
Hypothesized partial mediation structure. Here, a dependent variable is affected by an independent variable indirectly through a mediating variable (path encompassing arrows 2 and 3) and directly (path of arrow 1). The intervening variable, i.e., the mediator, mediates the indirect relationship. If the structure does not include arrow 1, then it is a full mediation structure.

Mediation analysis explains the mechanism by which one variable (*X*) affects another (*Y*) through a mediator (6). For example, the basic mediation structure depicted in Fig. 2 assumes a three-variable system such that there are two paths connecting the independent variable (*X*) to the dependent variable (*Y*). First, an indirect pathway (arrows 2 and 3) shows the indirect effect of *X* on *Y* through the mediator *Z*. The indirect pathway captures the independent variable affecting the mediator and the mediator, in turn, affecting the dependent variable. Second, the direct pathway (arrow 1) shows the direct effect of *X* on *Y*. One can consider the mediating variable capturing a mechanism through which an independent variable (X) impacts a dependent variable (*Y*), although we stress that mediation analysis does not necessarily establish causality (see the supplemental material on limitations of mediation analysis). In one hypothesized model within this mediation structure, the independent variable *X* impacts the dependent variable *Y* indirectly through the mediating variable as well as directly. This model is called the partial mediation model, as the mediator variable only partially mediates the effect of *X* and *Y*, and there also exists the direct effect of *X* on *Y*, besides the mediated effect through the mediator. An alternative model may show the independent variable impacting the dependent variable exclusively through the mediating variable without the direct pathway from *X* to *Y*. This model is called the full mediation model, as the mediator fully mediates the effect of *X* on *Y*, and, after controlling for this mediation effect, there is no direct effect of *X* on *Y*.

For example, we can use mediation analysis to investigate the mediating impact of a student’s prosocial utility value beliefs of biology (using biology to fulfill goals of helping others) as a mediator for the effect of using a textbook with prosocial examples (or a neutral control; the independent variable) on a student’s interest in the topic of biology (dependent variable) (7). We hypothesize that the textbook condition (prosocial or control) predicts interest through prosocial utility value beliefs. In other words, using a textbook with prosocial examples increases students’ belief in the social utility value of biology, and this increase, in turn, increases students’ interest in the topic of biology. The extent that students’ prosocial utility value beliefs of biology mediate the effect of textbook condition on student interest in biology can be estimated. If the mediation has a nonsignificant *P* value, or a significant *P* value but the estimated effect is small, we conclude that the effect of textbook condition on student interest in biology operates mostly through mechanisms other than a student’s prosocial utility value beliefs. If the size of mediation is large, a student’s prosocial utility value belief is more likely an important mediator for the effect of textbook condition on student interest. In this case, a student reading a prosocial textbook may lead to higher prosocial utility value beliefs, which, in turn, leads to higher interest in biology. If, after controlling for the mediating effect of prosocial utility value beliefs in biology, textbook condition still has a significant direct effect on student interest, this would be an example of a partial mediation model. Otherwise, it is a full mediation model.

## USING SEM FOR MEDIATION ANALYSIS

Structural equation modeling (SEM) is one powerful tool for mediation analysis. SEM is a broad approach to multivariable statistical analysis that explores structural relationships between number of variables (8). SEM uses a hypothesized set of linked regression equations to capture the structure of relationships among a web of latent and observed variables (6). Latent variables are those that cannot be observed or quantified; rather, values are inferred based on related observed variables that can be directly measured (9, 10). Examples include intelligence and science identity. Observed variables are those that can be observed and directly measured, such as survey responses and time spent on a task. While one could conduct mediation analysis with a series of regression models, there are a number of advantages to conducting mediation analysis with SEM (see the supplemental material on the use of SEM for mediation analysis).

Whereas regression analyses can fit one equation at a time to a data set, SEM allows us to simultaneously fit a set of regression equations to data and test how well those equations collectively capture the variation in the data. The set of regression equations captures a hypothesized structure of relations among variables, e.g., mediation structure, that link predictors to outcomes. Common software used to run SEM include the *lavaan* package in R (11), AMOS in SPSS (8), the CALIS procedure in SAS (12), and Mplus (13). Our examples of mediation analyses and corresponding syntax with SEM use *lavaan* package in R because of its intuitive syntax, particularly for those who are familiar with regression analysis in R. Another benefit of using *lavaan* is that the package, and other R resources, is open source and free, with extensive resources available online (for example, see http://lavaan.ugent.be/ and https://cran.r-project.org/web/packages/lavaan/lavaan.pdf).

## IDENTIFY A MEDIATION STRUCTURE

In multivariable statistical analysis, researchers consider alternative relationships between many variables to explain one or more outcome variables. A mediation structure consists of unidirectional hypotheses, depicted as pathways that go in a single direction between variables (14): the independent variable(s) affecting the mediator(s) and the mediator(s), in turn, affecting the dependent variable(s). Using data with some temporal element can help establish clear precedence in the direction of variable relationships (15). This also helps with decisions about which variables are independent and dependent and which ones can be mediators. In fact, some argue mediation analysis should be constrained to specific circumstances where the time-ordered relationship of variables is justifiable (16). For example, how does incoming preparation (such as entrance exam scores or high school grade point average) impact precourse science self-efficacy, which affects class performance? In this example, there is a clear temporal pattern, which makes the question suitable for mediation. Others believe one can also decide on the direction of pathways in a mediation structure based on theory (17). For example, if students take a precourse survey, two constructs included in that survey might gauge intrinsic motivation and science self-efficacy. An interesting question one might want to address is how intrinsic motivation and science self-efficacy interact, which, in turn, impacts performance. Unless there is strong theoretical rationalization underlying directionality, mediation analysis cannot address how these two constructs impact one another, as there is no temporal order for the two constructs.

## EVALUATE THE MEDIATION STRUCTURE

With the same set of variables, more than one mediation structure can be fitted to a data set. Fit statistics are used to evaluate mediation structure and identify possible mediators and mediation paths. SEM fit indices determine how well a hypothesized SEM model with a given set of mediators and mediation paths represents the observed data. One can compare fit indices of different SEM models to evaluate which model is a better fit for the data. Table 1 summarizes fit indices commonly used in SEM analyses based on references 8 and 18.

If a SEM model cannot be identified as a suitable fit, one should closely examine the model structure to decide which paths to exclude or add to the model to improve its fit. Theoretical considerations are warranted in such model revision processes, and only paths with existing theoretical justification should be considered (19). In the supplemental material, we describe steps to improve model fit through model revision in *lavaan*.

## PARTIAL VERSUS FULL MEDIATION MODEL ANALYSES

**FIG 3:**
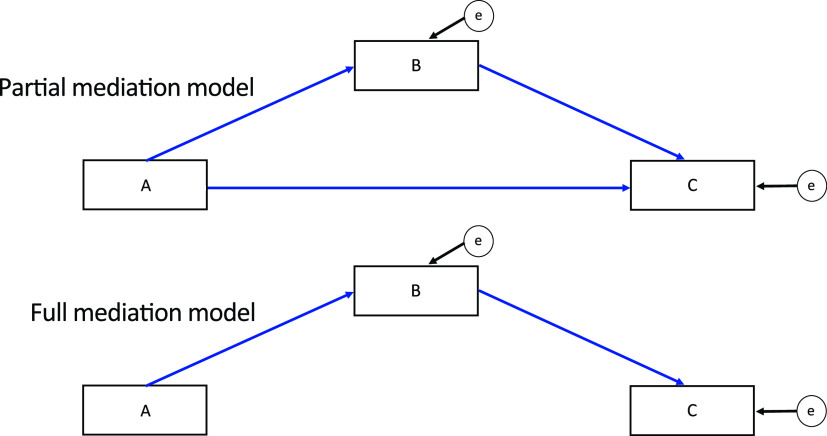
Contrasting partial and full mediation models to test the mediating effect of variable B in the relationship between variable A and variable C. The partial mediation model (top) tests the partial mediation effect of A on C. In this model, A directly affects C (i.e., A→C) and indirectly via B (i.e., A→B→C). The full mediation model (bottom) tests how B fully mediates the relationship between A and C (i.e., only A→B→C). The encircled “e” acknowledges error inherent to model estimates. Variables can be either observed (rectangles) or latent (ellipses).

Besides identifying the mediators and mediation paths, to finalize the mediation structure, one should decide on whether a partial or full mediation model is more appropriate in an analysis (Fig. 3). Fit statistics can be used for this purpose. If you are examining the mediating effect of B in the association A→B→C, a partial mediation model is one in which B only partially mediates the effect of A on C, and A impacts C indirectly via B as well as directly (Fig. 3). In a full mediation model, on the other hand, B fully mediates the relationship between A and C, and A impacts C only indirectly via B.

One can start by fitting a partial mediation model to the data. If this model is a good fit for the data, we expect that fit indices will fall within the acceptable range. If the direct link between the independent variable and the dependent variable is not significant, this suggests that a full mediation, rather than a partial one, can be used to analyze the data. Therefore, one can next fit the more parsimonious full mediation model to the data and compare the fit of the two mediation models. As a full mediation model is nested within the partial mediation model, chi-squared test of differences can be used to compare the chi-squared statistics of the two models. If the fit of the more parsimonious full mediation is better or similar to the more complex partial mediation model, then the full mediation model should be used for analysis. Other fit indices can also be used to compare the models by examining which model has more acceptable fit indices.

On the other hand, if the direct link between the independent variable and the dependent variable is significant and/or the partial mediation model is a better fit for the data, then the mediation is partial, and this more complex model should be used for data analysis. If the partial mediation model does not meet the fit index criteria and excluding the direct link between independent and dependent variables also does not address the issue, then additional steps can improve the overall structure of the model (see the supplemental material on steps to revise your model and improve model fit). Following these steps, one can return to the questions of whether the model should be partial or full mediation.

## INTERPRET AND VISUALIZE THE RESULTS

Once the appropriate model is identified and analyzed, reporting outcomes in ways that are easy to interpret for your audience is the next step of mediation analysis. Because mediation analysis includes different relations between variables and understanding all those relations can be cognitively taxing, using visualizations can be helpful. In common visualizations, each link represents a relationship between two variables. The estimated coefficient (*b*), the standard error of the coefficient (SE), and the *P* value of significance (*p*) of the link can be reported above or below the link. Visualizations depict different paths and their sizes in the model (see example in the supplemental material for results overlaid on visualization).

The estimated coefficient (*b*) communicates the magnitude of the effect that one variable has on another, i.e., the strength of the link between two variables. For example, in Fig. 3, if the reported coefficient for the A→B relationship is *b* = 0.80, one would conclude that for every unit increase in A, B would increase by 0.80 units. If *b* = −0.80, one would conclude that for every unit increase in A, B decreases 0.80 units. The standard error of the coefficient reflects the variance of this estimated effect in a sampling distribution.

In order to make results more interpretable for readers, we recommend normalizing continuous variables that do not have meaningful units. For example, the measurement units for latent social psychological constructs are quite arbitrary, and it is hard to interpret what one unit increase means for such variables unless we know the overall distribution of the sample. By normalizing these variables, the measurement unit is relative to the sample distribution (e.g., students in a classroom). In other words, model coefficients can be interpreted in terms of standard deviations from a mean value. In *lavaan*, one can use the standardizing feature of the package to normalize latent continuous variables and use the coefficients reported in the Std.lv output column. If both latent and observed variables are continuous and all should be normalized, then one can report the coefficients in the Std.all output column.

## CONCLUSIONS

Many questions addressed in higher education research explore underlying mechanisms for academic outcomes. Because students and learning environments are multifaceted and complex, more than one variable will contribute to outcomes of interest. Therefore, researchers will profit from an introduction to statistical methods that explicitly address relations among multiple variables and outcomes. Mediation analysis is a powerful tool for this purpose. Mediation analysis examines whether the effect of an independent variable on a dependent variable is mediated by a third variable, i.e., a mediator. To conduct mediation analysis, we recommend SEM that, unlike regression analysis, allows multiple regression models to be fitted to data simultaneously and tests how they collectively capture variation in the data set. Although SEM has limitations (see the supplemental material on limitations of mediation analysis), it is a valuable approach for exploring relationships in multivariable analyses across education research fields. While mediation analysis can be conducted using approaches other than SEM, and while SEM can be applied to many statistical problems beyond mediation analysis, we focused specifically on mediation analysis in discipline-based education research using this approach. In doing so, we hope to provide an accessible introduction for education researchers to mediation analysis as they continue to examine complex problems in higher education.
