# Supplementary material for: Mediation Analysis in Discipline-Based Education Research Using Structural Equation Modeling: Beyond “What Works” to Understand How It Works, and for Whom
Source: J Microbiol Biol Educ. 2021 Jun 30;22(2):e00108-21. doi: 10.1128/jmbe.00108-21 (PMC8442014; doi:10.1128/jmbe.00108-21)
Supplement: SUPPLEMENTAL FILE 1 — Download JMBE00108-21_Supp_1_seq1.docx, DOCX file, 1.5 MB [file jmbe00108-21_supp_1_seq1.docx]

**Supplementary Materials**

**Mediation analysis in discipline-based education research using structural equation modeling: beyond “what works” to understand how it works, and for whom**

Cissy J. Ballen^1*^ and Shima Salehi^2^

^1^Department of Biological Sciences, Auburn University, Auburn, AL

[^*^mjb0100@auburn.edu](about:blank)

^2^Graduate School of Education, Stanford University, Stanford, CA

**Supplemental materials include:**

*Why use SEM for mediation analysis?*

*Table S1.* Summary of vocabulary used in structural equation modeling (SEM) and other quantitative research from the social sciences

*Steps to revise your model and improve model fit*

*Example from the literature:* introductory science as an entryway or conveyor belt?

*Limitations of mediation analysis*

*References*

***Why use SEM for mediation analysis?***

Instead of using SEM, one could alternatively conduct a series of regression models to conduct mediation analysis, which may be a more familiar technique for some researchers. For example, in the mediation structure presented in Figure 2 in the main text, one can conduct mediation analysis by running the following three regression models one at a time:

$$Model 1: Y= \gamma_{1}+ cX+\varepsilon_{1}$$

$$Model 2: M= \gamma_{2}+ a X+\varepsilon_{2}$$

$$Model 3: Y= \gamma_{3}+ c^{'}X+b M+ \varepsilon_{3}$$

In these regression models, c is the total effect size of X on Y, a*b is the effect size of the mediation, and c’ is the direct effect size of X on Y. In order to evaluate the significance of indirect effects of X on Y through a mediator (a*b), one must use additional tests such as the joint significance test (1), Sobel test (2), or bootstrapping (a non-parametric analysis for non-normal distributions; 3, 4).

Compared to conducting mediation analysis with a series of regression models, there are a number of advantages to conducting mediation analysis with SEM. First, a single SEM model can be used to examine multiple different mediation paths simultaneously and address missing data along each path. Second, SEM has a built-in function to estimate the effect size and significance of each mediation path, which is easier than using ad hoc methods of calculation required in regression methods (5, 6, 7).Third, SEM is particularly powerful when latent variables (measured by multiple items) are included in the analysis. For example, someone could investigate confidence as a mediator for the effect of binary gender (independent variable) on test performance (dependent variable). In this example, confidence is a latent variable measured with multiple survey items. In such cases, SEM can account for the measurement error of the latent variable through confirmatory factor analysis (CFA) (8). Fourth, SEM provides fit indices to test how well the overall hypothesized mediation structure fits the data. All of these features are not possible with regression analysis.

**Table S1.** Summary of vocabulary used in structural equation modeling (SEM) and other quantitative research from the social sciences.

|  | Definition | Example(s) |
| --- | --- | --- |
| Latent variables | ‘Hidden’ variables cannot be observed or quantified. Values are inferred based on related observed variables that can be directly measured (11, 12). | Intelligence, science self-efficacy, science identity. |
| Observed variables | Variables that can be observed and directly measured. | Standardized rating scales, survey responses, time spent on a task. |
| Exogenous variable | Independent variables in SEM equations. | Gender, minority status, first generation status (see Figure S1). |
| Endogenous variable | Variables that serve as a dependent variable in at least one SEM path. They are described as an ‘endogenous’ rather than ‘dependent’ variables because they may also act as an independent variable in other paths (5). | All variables that are affected by other variables are endogenous variables. In Figure S1, these include incoming preparation and exam performance. |
| Mediating variable | The variable through which an independent variable impacts a dependent variable (1, 13) | In Figure S1, we show incoming academic preparation serves as a mediating variable for the effect of first-generation status on exam performance in introductory science courses. |
| Direct effect | The effect of an independent variable on a dependent variable (1, 14). | The left side of Figure S1 shows a hypothesized mediation model in which demographic status affected exam performance directly. |
| Indirect effect | The effect of an independent variable on a dependent variable through a mediating variable (1, 14). | Figure S1 shows hypothesized mediation models in which exam performance is affected by the demographic status *indirectly via incoming preparation.* |
| Full mediation | Occurs when the relationship between the independent variable and dependent variable is fully captured by the indirect effect via a mediating variable (1, 14). | The right side of Figure S1 shows a hypothesized full mediation model in which incoming preparation mediates the relationship between the demographic status variables and exam performance. In this case, exam performance is affected by the demographic status only *indirectly* through incoming preparation. |
| Partial mediation | Occurs when the indirect effect via a mediating variable accounts for some, but not all, of the relationship between the independent variable and dependent variable. A partial mediation model includes both indirect and direct effects between the independent and dependent variables (1, 14). | The left side of Figure S1 shows a hypothesized partial mediation model in which incoming preparation partially mediates the relationship between the demographic status variable and exam performance. In this case, exam performance is affected by the demographic status *indirectly* through incoming preparation, as well as *directly*. |

***Steps to revise your model and improve model fit***

In lavaan, one can pursue the following three steps to improve the model fit: 1) identify the problem by examining the residuals matrix of the model. The residual matrix is the observed covariance matrix subtracted by the fitted covariance matrix. One can access the residual matrix *“print(resid(mediation.model))”* in lavaan. The residual matrix is presented as correlational units, and usually items with an absolute value greater than 0.1 should be examined further. If there is a high covariance residual for any two variables in the model, that suggests those two variables are highly correlated and hence adding a path between the two in the model to be freely estimated might improve the model fit; 2) Extend the model examination by using Modification Indices, *print(modindices((mediation.model))*, to examine which path should be added to the model. The higher the Modification Index of a path, the more its addition improves the model; 3) exclude nonsignificant path(s) if the exclusion does not hurt the model fit indices. Nonsignificant paths can be identified in the model output results.

***Example from the literature: introductory science as an entryway or conveyor belt?***

Here we provide an example using real data related to performance outcomes based on student demographic information. We selected this example because it is relatively simple, with few variables, but also acknowledges the enormous potential for complexity, which is a strength of mediation analysis.

Gaps in academic performance can sometimes be predicted by categorical descriptors of identity (e.g., gender, minority status, age, major; 14, 15). The problem with explanations based on these classifications is they oversimplify the mechanisms that influence outcomes, and provide little insight on how to address them. For example, students arriving from low-socioeconomic status school districts might struggle with academic content due to wide gaps in incoming academic preparation (17). Thus, we set out to examine one underlying mechanism that impacts performance as we approached a sizeable data set spanning across multiple large introductory STEM classes at a midwestern university (e.g., biology, physics, chemistry; N = 2871). We aimed to explicitly test the relationship between (1) incoming preparation as measured by standardized entrance exams, (2) demographic traits such as whether the student is the first generation in their family to attend college (Fgen), their underrepresented minority (URM) status^[[1]](#footnote-1)^, and their binary gender (based on institutional information), and (3) student exam performance during introductory STEM classes in higher education.

First, we identified a mediation structure that had a clear temporal justification. We hypothesized that demographic traits affect exam performance through the mediating impact of incoming preparation (Figure S1). Second, based on fit statistics, we addressed whether a partial or full mediation model was more suitable for our data set (Figure S1, Figure S2). In one scenario, incoming preparation fully mediated the relationship between demographic traits and performance; in this scenario, e.g., first generation students may enter university with lower incoming academic preparation, and those measures predict their performance. In another scenario, even after considering the mediating impact of incoming preparation, demographic traits may still predict exam performance. This suggests other unidentified variables that relate to these broad facets of identity are also impacting student performance.

**Figure S1.** Contrasting partial and full mediation models to test the mediating effect of incoming preparation in the relationship between demographic categories - such as first generation college-going status (Fgen), underrepresented minority status (URM), and gender – and exam performance. The partial model (left) tests the partial mediation effect of Fgen + URM + gender on Exam performance. In this model, Fgen + URM + gender directly affects exam performance, as well as indirectly via incoming preparation. The full mediation model (right) tests whether incoming preparation fully mediates the relationship between Fgen + URM+ gender and Exam performance. For example, in this model, a demographic variable predicts student incoming preparation, which predicts exam performance.


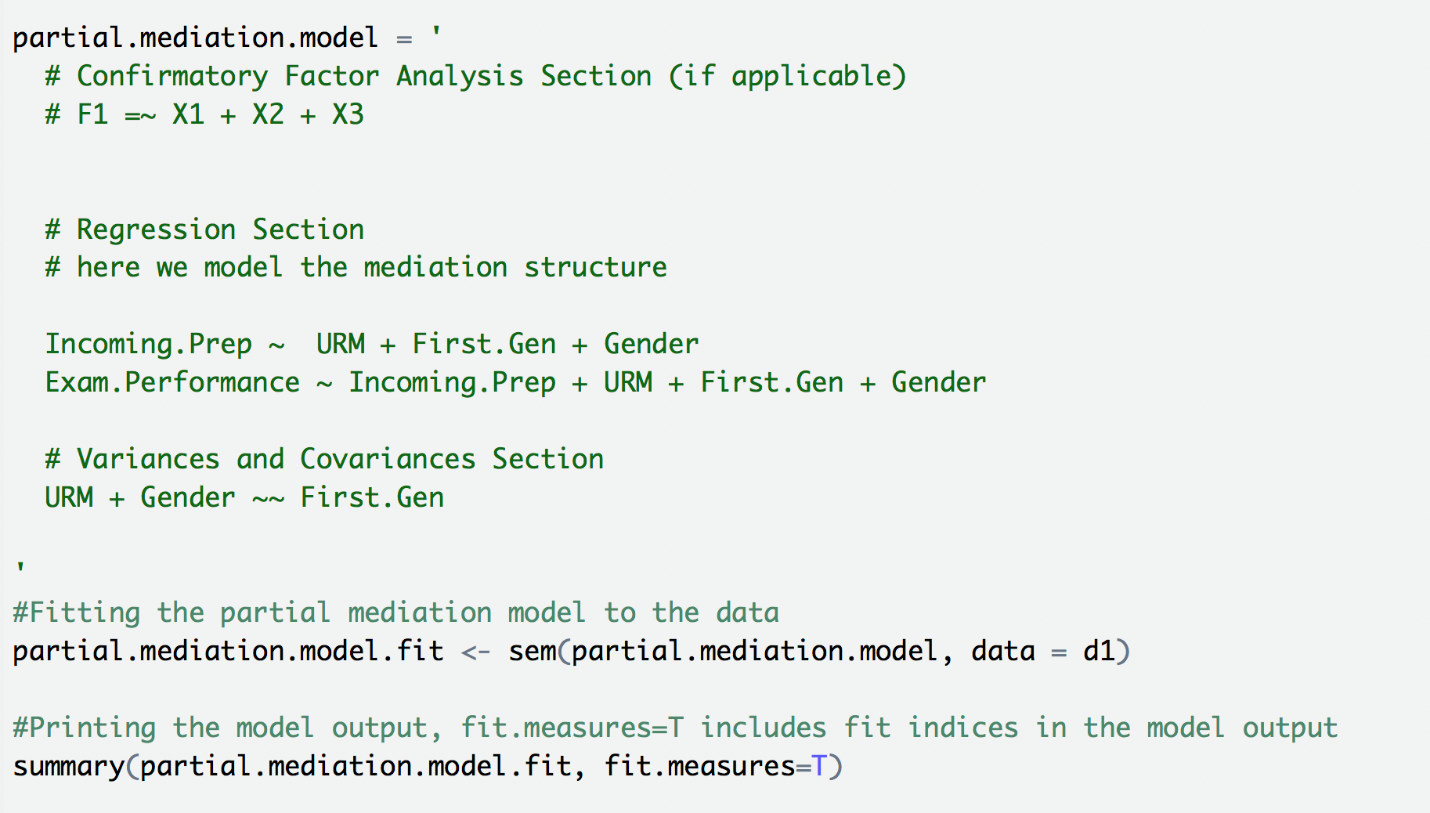


**
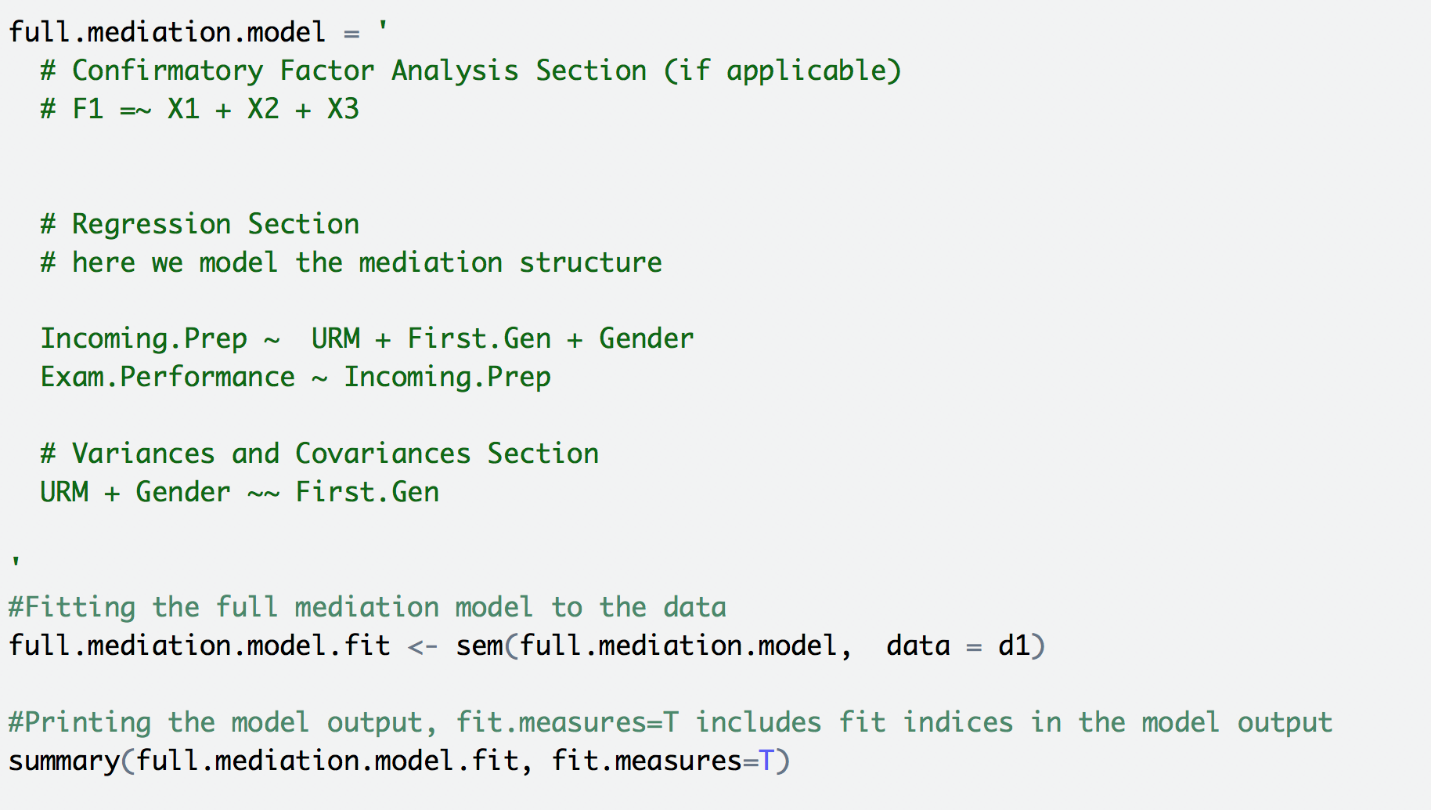
**

**
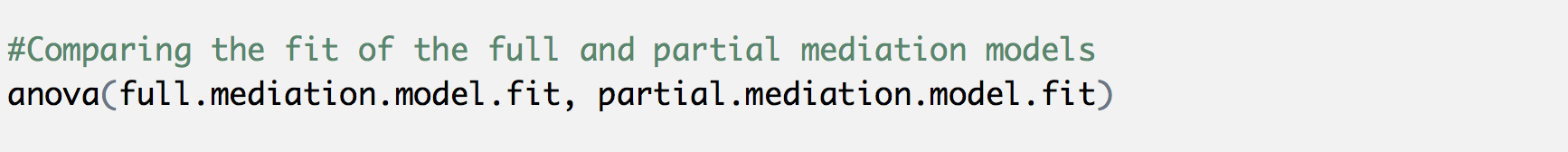
**

**Figure S2.** Annotated output fitting (a) partial and (b) full mediation models to the data and then (c) comparing their fit. First, for the partial mediation model, dependent variables include incoming preparation and exam performance. In this model, demographic factors (first generation status, URM and gender) predict incoming preparation, and demographic factors and incoming preparation predict exam performance. In the full mediation model, demographic factors predict incoming preparation, which in turn predicts exam performance. Finally, we specify the variance and covariance of variables that we want to be freely estimated, e.g. covariance of independent variables. In this example, we have included the covariances of URM, gender, and first generation. By doing this, the model estimates the covariance between URM status and first-generation status, as well as the covariance between gender and first-generation status. The variance and covariance section can be used to specify relationships that are not important for the estimated structure of the model but estimating them can improve the fit of the model. After defining the model, we fit the model to the data (d1). Then, we print out the results of the fitted model. We add “fit.measures=T” for the fit indices to be included in the model output.

We found that the partial mediation model was a better fit for the data (X2(3) = 22.258, *P* <0.0001). In other words, demographic status directly predicted exam performance, as well as indirectly through incoming preparation (a mediating variable). The indirect relation shows first generation, URM, and women students entered introductory science classes receiving less prior-preparation (i.e., they had lower entrance exam scores), which predicted how they performed in those classes. For the purposes of this example, we only tested the mediating effect of incoming preparation, but there are many other mechanisms that affect the performance of students. After controlling for the mediating effect of incoming preparation, significant gaps in performance persisted among different demographic groups. This is reflected by the direct effect of demographic status on exam performance, which is included in the model. While our work on this student population has started to elucidate those other hidden variables (18), future work across institutions will profit from the use of mediation analysis in such investigations. The fit indices of the partial mediation model fell within the acceptable range: CFI: acceptable range above 0.95, RMSEA: acceptable range 0-0.07; SRMR: acceptable range 0-0.1 (Figure S3).

We analyzed the impact of URM, Fgen, and binary gender on normalized incoming preparation (Figure S3). When interpreting outcomes, the ‘Estimate’ shows the difference between categorical groups (Figure S3). For example, it shows that in our sample, URM students on average score 0.63 standard deviation lower than non-URM students (*P* < 0.001) on the ACT. This demographic, along with first-generation students and women, all enter college with significantly lower entrance exam scores. Next, our results show relationships between variables and normalized exam performance, or how students perform on exams relative to others in their class who took the same exams. The partial mediation model in Figure S1 shows how we tested for the effect of demographic variables (URM, Fgen, and binary gender) as well as the mediating effect of incoming preparation. Our results show that incoming preparation has the largest impact on exam performance in introductory courses; for example, one standard deviation increase in incoming preparation leads to 0.37 standard deviation increase in exam performance. In other words, students who entered higher education more prepared have a substantial ‘leg up’ in introductory courses.

**Figure S3.** Results from the analysis in our example can be interpreted by readers as they visualize the structure of the partial mediation model. Variables from the results are color-coded as they correspond to pathways in the model (blue, red, orange).

We found demographic descriptors directly impact exam performance in introductory biology, as well as indirectly through their impact on incoming preparation. Overall, incoming preparation strongly predicts how students perform on exams in introductory classes.

***Limitations of mediation analysis***

Mediation analysis has a number of limitations that warrant consideration. Here we focus on three of the most significant limitations related to causality, unidirectional constraints, and sample size. First, researchers must make important distinctions regarding causality when interpreting results from mediation analysis. A causal mechanism is considered the *process* through which a variable influences an outcome (19, 20). Approaches that rely on structural equation modeling do not necessarily meet assumptions required to identify a causal variable in mediation. To conduct causal mediation analysis, the *mediation* package in R (21) is particularly designed to enable causal mediation based on experimental data (19).

Second, a limitation to this approach is that mediation analyses are unidirectional hypotheses. This means that the pathways can go in a single direction between two variables (22). To address this issue, we recommend collecting data with some temporal element, where one can establish precedence in the order of variable relationships (23). While some researchers apply mediation analyses to cross-sectional research designs, this requires strong theoretical justification (23).

A limitation for some investigators is the sample size requirement to run mediation analysis with SEM. A strength of SEM is its flexibility and ability to examine complex relationships among variables across alternative models. But these same features call for relatively large sample sizes; the more parameters included in the model, the larger the sample size required for robust analysis. It is important to consider whether your sample size is adequate for achieving statistical power in order to observe true relationships in the data (24). Estimates such as standard error accuracy may be compromised if the sample data is too small (6). A small sample can also lead to an underpowered model. When a model is underpowered, it is less probable that the null hypothesis will be rejected. This is particularly an issue in SEM analysis, in which the null hypothesis means that the fitted model created by the researcher is consistent with the data; and rejecting the null hypothesis is rejecting the researcher’s model. Therefore, with low power, there is the higher risk of accepting the researcher’s model when it is not a good fit to the data (6). This differs from conventional hypothesis-testing (e.g., t-test), in which the alternative (rather than null) hypothesis supports the researcher’s model. In this case, rejecting the null hypothesis is accepting the researcher’s model. Therefore, when the analysis is underpowered, it is less probable to accept the researcher’s model.

**References**

1. Baron RM, Kenny DA. 1986. The moderator–mediator variable distinction in social psychological research: Conceptual, strategic, and statistical considerations. J Pers Soc Psychol 51:1173.

2. Sobel ME. 1982. Asymptotic confidence intervals for indirect effects in structural equation models. Sociol Methodol 13:290–312.

3. Hesterberg T, Moore DS, Monaghan S, Clipson A, Epstein R. 2005. Bootstrap methods and permutation tests. Introd to Pract Stat 5:1–70.

4. Mooney CF, Mooney CL, Mooney CZ, Duval RD, Duvall R. 1993. Bootstrapping: A nonparametric approach to statistical inference. Sage.

5. Gunzler D, Chen T, Wu P, Zhang H. 2013. Introduction to mediation analysis with structural equation modeling. Shanghai Arch psychiatry 25:390.

6. Kline RB. 2015. Principles and practice of structural equation modeling. Guilford publications.

7. MacKinnon D. 2012. Introduction to statistical mediation analysis. Routledge.

8. Knekta E, Runyon C, Eddy S. 2018. One size doesn’t fit all: Using factor analysis to gather validity evidence when using surveys in your research. CBE Life Sci Educ 18:1–17.

9. Iacobucci D, Saldanha N, Deng X. 2007. A meditation on mediation: Evidence that structural equations models perform better than regressions. J Consum Psychol 17:139–153.

10. Knekta E, Runyon C, Eddy S. 2019. One Size Doesn’t Fit All: Using Factor Analysis to Gather Validity Evidence When Using Surveys in Your Research. CBE—Life Sci Educ 18:rm1.

11. Spearman C. 1904. “ General Intelligence,” objectively determined and measured. Am J Psychol 15:201–292.

12. Bollen KA. 2014. Structural equations with latent variables. John Wiley & Sons.

13. Collins LM, Graham JJ, Flaherty BP. 1998. An alternative framework for defining mediation. Multivariate Behav Res 33:295–312.

14. Rucker DD, Preacher KJ, Tormala ZL, Petty RE. 2011. Mediation analysis in social psychology: Current practices and new recommendations. Soc Personal Psychol Compass 5:359–371.

15. Ballen CJ, Mason NA. 2017. Longitudinal Analysis of a Diversity Support Program in Biology: A National Call for Further Assessment. Bioscience 67:367–373.

16. Koester BP, Grom G, McKay TA. 2016. Patterns of gendered performance difference in introductory STEM courses. arXiv Prepr arXiv160807565.

17. Alexander C, Chen E, Grumbach K. 2009. How leaky is the health career pipeline? Minority student achievement in college gateway courses. Acad Med 84:797–802.

18. Ballen CJ, Salehi S, Cotner S. 2017. Exams disadvantage women in introductory biology. PLoS One 12:e0186419.

19. Imai K, Keele L, Tingley D, Yamamoto T. 2011. Unpacking the black box of causality: Learning about causal mechanisms from experimental and observational studies. Am Polit Sci Rev 105:765–789.

20. Hicks R, Tingley D. 2011. Causal mediation analysis. Stata J 11:605–619.

21. Tingley D, Yamamoto T, Hirose K, Keele L, Imai K. 2014. Mediation: R package for causal mediation analysis.

22. Tomarken AJ, Waller NG. 2005. Structural equation modeling: Strengths, limitations, and misconceptions. Annu Rev Clin Psychol 1:31–65.

23. Kline RB. 2015. The mediation myth. Basic Appl Soc Psych 37:202–213.

24. Wolf EJ, Harrington KM, Clark SL, Miller MW. 2013. Sample size requirements for structural equation models: An evaluation of power, bias, and solution propriety. Educ Psychol Meas 73:913–934.

1. Characterizing individuals with Underrepresented minority (URM) status is not clear-cut, and some thoughtfully disagree with the classification altogether. For the purposes of this analysis, URM is defined as a group whose representation in science is lower than their representation in the country. These individuals include Hispanic/Latinx, African Americans, Native Americans, Native Hawaiian/Pacific Islanders, and those of two or more races. [↑](#footnote-ref-1)
